# Antibiotic pretreatment minimizes dietary effects on reconstructure of rumen fluid and mucosal microbiota in goats

**DOI:** 10.1002/mbo3.537

**Published:** 2017-10-05

**Authors:** Hong Shen, Zhongyan Lu, Zhihui Xu, Zanming Shen

**Affiliations:** ^1^ College of Life Science Nanjing Agricultural University Nanjing Jiangsu China; ^2^ Bioinformatics Center Nanjing Agricultural University Nanjing Jiangsu China; ^3^ Key Lab of Animal Physiology and Biochemistry College of Veterinary Medicine Nanjing Agricultural University Nanjing Jiangsu China

**Keywords:** biodiversity, cell‐microbe interactions, gut bacteria, stress response

## Abstract

We used 16S *rRNA* gene sequencing to examine the posteffects of antibiotic treatment on the structure and metabolism of rumen microbiota. Twelve goats were randomly assigned into two groups, with one group receiving intramuscular streptomycin injection at 40 mg/kg bodyweight daily for 10 days. At 4 weeks after treatment with antibiotic, three goats were randomly selected from each group and switched to a 35% concentrate diet. The remaining six goats continued with the 10% concentrate diet. At 4 weeks after dietary shift, ruminal fluid and epithelium were collected to analyze the microbiota composition and short‐chain fatty acid (SCFA) concentrations of the rumen. We found that antibiotic administration led to increases in the diversity and richness of recovered mucosal microbiota and to decreases in those of recovered fluid microbiota. When dietary modulation was performed after antibiotic intake, both communities showed little difference in structure from premodulated states. Additionally, antibiotic pretreatment reduced the basal lines of individual SCFAs but did not affect the increased percentages of SCFAs. Overall, our results indicate that antibiotic administration affects the structure of both rumen fluid and mucosal microbiota and reduces the functional redundancy of rumen microbiota.

## 1 INTRODUCTION

The rumen is inhabited by a vast ensemble of symbiotic microbes (the microbiota), involving bacteria, methanogenic archaea, ciliate protozoa, and anaerobic fungi. Previous studies showed that bacteria, protozoa, and fungi took roles in the digestion of cellulose‐rich feeds and conversion of them into the short‐chain fatty acids (SCFAs), which meet approximately 80% of the energy requirement of the whole body (Bergman, 1990). Archaea is necessary for the proceeding of the overall ruminal fermentation, through the removal of reducing equivalents in the rumen (Krause, Nagaraja, Wright, & Callaway, 2013). Researchers identified that bacteria occupied the dominant place in numbers and also contributed greatest to the rumen fermentation within the microbiota. Because of the local oxygen content and the type of available carbohydrates, heterogenetic bacterial communities exist in the mucosal layer and the luminal content of the rumen (Wetzels et al., 2015; Wetzels et al., 2015). Studies of gastrointestinal (GI) microbiota have suggested that the role of microbial communities varies with their spatial distribution within the GI tract (Gevers et al., 2014; Hold & Garrett, 2015; Lavelle et al., 2015). The mucosa‐associated community, which intimately contacts the GI epithelium, seems to affect the expression of the host genes that regulate nutrient uptake and the metabolism of the GI epithelium (Hooper & Gordon, 2001; Malmuthuge et al., 2012). On the other hand, the luminal community, which is directly in contact with the food particles, is thought to affect fermentation and the generation of energy substrates in the GI tract (Mao, Huo, & Zhu, 2016). However, rather than isolated communities, they cooperated with each other in such as keeping H_2_ gas level and recycling of *N* to maintain the rumen homeostasis (Cheng, McCowan, & Costerton, 1979; Nava, Carbonero, Croix, Greenberg, & Gaskins, 2012). Furthermore, they developed cooperative mechanisms such as horizontal gene transfer, biofilm formation, and quorum sensing to maintain their own niches (Ohland & Jobin, 2015). Disturbance of any of these communities might therefore affect the balance of the GI microbiota and, concomitantly, predispose the host to the infections and diseases (Ohland & Jobin, 2015).

Antibiotics are traditionally used for disease prevention and growth promotion in ruminant animals. Substantial evidence has shown that antibiotic use can induce an immediate reduction in the GI microbiota (Antonopoulos et al., 2009; Jernberg, Lofmark, Edlund, & Jansson, 2007). After the ceasation of antibiotic administration, the GI microbiota can recover within a few days to a few weeks (De La Cochetière et al., 2005; Dethlefsen, Huse, Sogin, & Relman, 2008). However, the composition of recovered microbiota is normally different from the pretreated state (Antonopoulos et al., 2009; Jernberg et al., 2007). Moreover, the antibiotic‐induced alteration of GI microbiota is suggested to have profoundly negative effects on the health and physiological functions of the host. For example, the antibiotic‐induced alteration of GI microbiota in childhood is associated with the appearance of chronic conditions such as asthma and atopic disease in humans (Dethlefsen et al., 2008). To date, the posteffects of antibiotic use on the spatial organization and fermentation function of rumen microbiota have not yet been reported.

Our previous studies have shown that dietary concentrate promotes microbial fermentation and energetic absorption in the goat rumen (Lu et al., 2015; Yan, Zhang, & Shen, 2014). In the present study, we first investigated the compositional recovery of rumen fluid and mucosal microbiota in goats undergoing a 10‐day streptomycin (ST) treatment. The posteffects of antibiotic use on rumen fermentation were estimated by comparing the responses of recovered rumen microbiota to the increase in dietary concentrate with the responses of that without exposure to the antibiotic. Our findings provided a better understanding of the spatial dynamics of the rumen microbiota in response to environmental stress. Moreover, microbial interactions and specific species within the indigenous microbiota response to disturbance may have value in predicting future instability and disease, and aid in managing the rumen microbial ecosystem.

## 2 EXPERIMENTAL PROCEDURES

**Figure 1:**
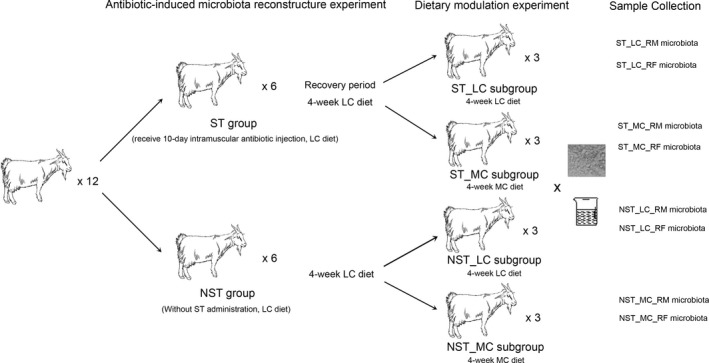
Experimental design applied in this study. Twelve goats were randomly assigned into two groups, with one group receiving intramuscular streptomycin injection of 40 mg/kg bodyweight daily for 10 days. Goats and rumen microbiota were given 4 weeks to recover from the antibiotic treatment. After that, three goats were randomly selected from each group and switched to a 35% concentrate diet. The remaining six goats continued with the 10% concentrate diet. After 4 weeks, ruminal fluid and ruminal epithelium were collected from all goat

Two experiments were successively conducted in this study (Figure 1). Both experiments were approved by the Animal Care and Use Committee of Nanjing Agricultural University, in compliance with the Regulations for the Care and Use of Animals (Nanjing Agricultural University, 1999).

### 2.1 Animal experiments

Twelve healthy goats (4‐month‐old, Boer × Yangtze River Delta White, 14–16 kg bodyweight) without prior exposure to antibiotics were used. All goats were fed with a pure hay diet ad libitum for 4 weeks before the start of the experiments to ensure adaptation to their new environment. The method of intramuscular injection was employed in the study to ensure that the same dose of streptomycin was received by the goats.

#### 2.1.1 Experiment 1 (antibiotic‐induced microbiota reconstruction experiment)

Twelve goats were fed with a diet containing 90% hay plus 10% concentrate (LC diet, Table S2) during the whole period of *experiment 1*. Goats were randomly assigned into two groups and treated with (referred to as the ST group, *n* = 6) or without (referred to as the NST group, *n* = 6) intramuscular injection of streptomycin (Merck, Shanghai, China) at the dose of 20 mg/kg bodyweight at 0700 h and 1600 h daily during the first 10 days of *experiment 1*. Subsequently, the goats and GI microbiota were given 4 weeks to recover from the streptomycin treatment. This period, which lasted for 38 days was followed by *experiment 2*.

#### 2.1.2 Experiment 2 (dietary modulation experiment)

Six goats in the NST group were randomly assigned into two groups. One group was continuously fed with the LC diet (referred to as the NST_LC group, *n* = 3), and the other (referred to as the NST_MC group, *n* = 3) was shifted to a diet consisting of 65% hay plus 35% concentrate (MC diet, Table S2). The same procedure was applied to the goats as in the ST group. Accordingly, one subgroup of ST group, which continued with the LC diet, was referred to as the ST_LC group in this study, and the other subgroup, which was fed with the MC diet, was referred to as the ST_MC group in this study. *Experiment 2* lasted for 28 days.

In the experimental period (66 days in total), all the goats were placed in individual pens (1.2 × 1.0 m) and fed with two equal portions of diet at 0800 and 1700 h daily. The composition of the MC and LC diets is presented in Table S2. Fresh water was freely available to all goats during the experiments.

### 2.2 Sample collection

On day 29 of the *experiment 2*, all goats were killed at a local slaughter house. Immediately after slaughter, approximately 20 ml ruminal fluid was strained through a 4‐layer cheese cloth and stored at −20°C for the determination of SCFA concentration and for the extraction of microbial DNA. Rumen tissue from the ventral blind sac was quickly excised and gently washed using ice‐cold phosphate‐buffered saline (PBS; pH 7.4). The epithelium of the ventral rumen sac was quickly separated from the tissue and cut into 1–2 cm^2^ pieces. One piece was placed on ice and promptly used for the extraction of microbial DNA.

### 2.3 Determination of SCFA concentrations

SCFA concentrations were determined using the chromatograph HP6890N (Agilent Technologies, Wilmington, DE, USA) as described by (Yang, Shen, & Martens, 2012). In brief, nitrogen (99.99% purity) was used as the carrier gas at a constant flow rate of 2.8 ml/min and a split ratio of 1:30. The capillary column temperature was set to 140°C for 4 min and then increased at 25°C/min to 240°C. The temperatures of the injection port and the flame‐ionization detector were set to 180°C and 250°C, respectively. Tiglic acid (C5H8O2) was used as an internal standard.

### 2.4 Microbial DNA extraction and sequencing

The metagenomic DNA of the rumen fluid microbiota was extracted from the ruminal fluid using a Bacterial DNA Kit (Omega, Shanghai, China) according to the manufacturer's protocol. The metagenomic DNA of the rumen mucosal microbiota was extracted from the mucosal layer of ruminal epithelium. To detach the bacteria from the RM, the ruminal epithelium was placed in 1.5 ml tubes with 0.7 ml PBS and several plastic beads and moderately shaken on a vortex for 30 s. The ruminal epithelium was transferred to a new tube and processed with the above step again. Subsequently, the microbial DNA on RM was extracted from the PBS mixture using the Bacterial DNA Kit according to the manufacturer's protocol.

The DNA concentration was determined using a Nanodrop 1000 (Thermo Fisher Scientific, Wilmington, DE, USA) and stored at −20°C until further processing. The 16S rRNA gene library was prepared using polymerase chain reaction (PCR) amplification of the V3–V4 region. The universal primers 338F (5'‐ACTCCTACGGGAGGCAGCAG‐3') and 806R (5'‐GGACTACHVGGGTWTCTAAT ‐3') (Mori et al., 2014), including TruSeq adapter sequences and indices, were used in the PCRs. All libraries were sequenced using an Illumina MiSeq platform (Illumina, San Diego, California, USA) at Biomarker Technologies, Beijing, China.

### 2.5 Rumen microbiota analysis

Paired reads were filtered for quality (Q30) and joined by FLASH version 1.2.11 (Magoc & Salzberg, 2011). Sequences that contained read lengths shorter than 400 bp were removed. The remaining sequences were then classified into taxa by blasting with the ribosomal database project (RDP) database at a 97% similarity threshold. Operational taxonomic units (OTUs), whose counts were more than 3 in at least one of the samples, were retained in the further analysis. The selected OTUs were normalized to the relative abundance for each sample. The diversity of the microbial communities was estimated using the R program phyloseq package (McMurdie & Holmes, 2013). The richness of the microbial communities was estimated using the dilution curve. For a deeper analysis of the diversity of the major evolutional clades in the microbial community, the microbial data were filtered to retain OTUs whose relative abundances were more than 1% in at least one sample. The complete 16S *rRNA* sequences of four archeaes were downloaded from NCBI, and used as the outgroups in the following analysis. MUSCLE *version* 3.8.31 (Edgar, 2004) was used to align the received 16S *rRNA* sequences of the OTUs and complete 16S *rRNA* sequences of four archeaes. RAxML *version 8* (Stamatakis, 2014) and the GTR model were used to construct the maximum likelihood trees. The R program ape package (Paradis, Claude, & Strimmer, 2004) was used to plot the tree.

### 2.6 Data submission

The metagenomic data are available at the NCBI under BioProject PRJNA349213.

## 3 RESULTS

### 3.1 Antibiotic induced long‐lasting alterations in rumen fluid and mucosal microbiota

**Figure 2:**
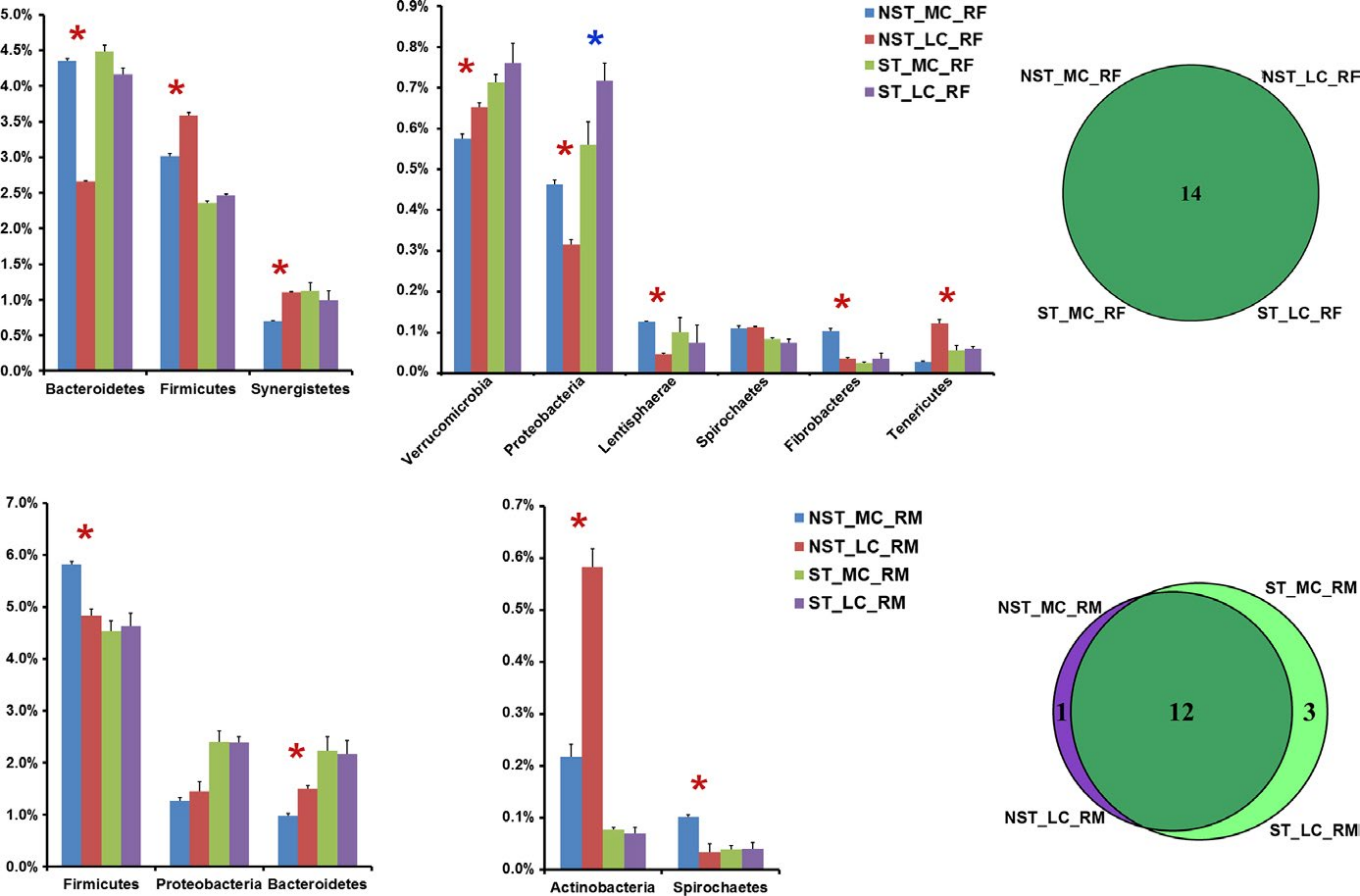
Phylum‐level comparison of rumen fluid (RF) microbiota and rumen mucosal (RM) microbiota between samples from goats without exposure to the antibiotic and samples from goats with antibiotic treatment. Only the detectable phyla (the relative abundance was more than 1% in at least one of the given samples) were present here. Star indicates that significant differences are observed in the *t*‐test (*p *<* *.05)

**Figure 3:**
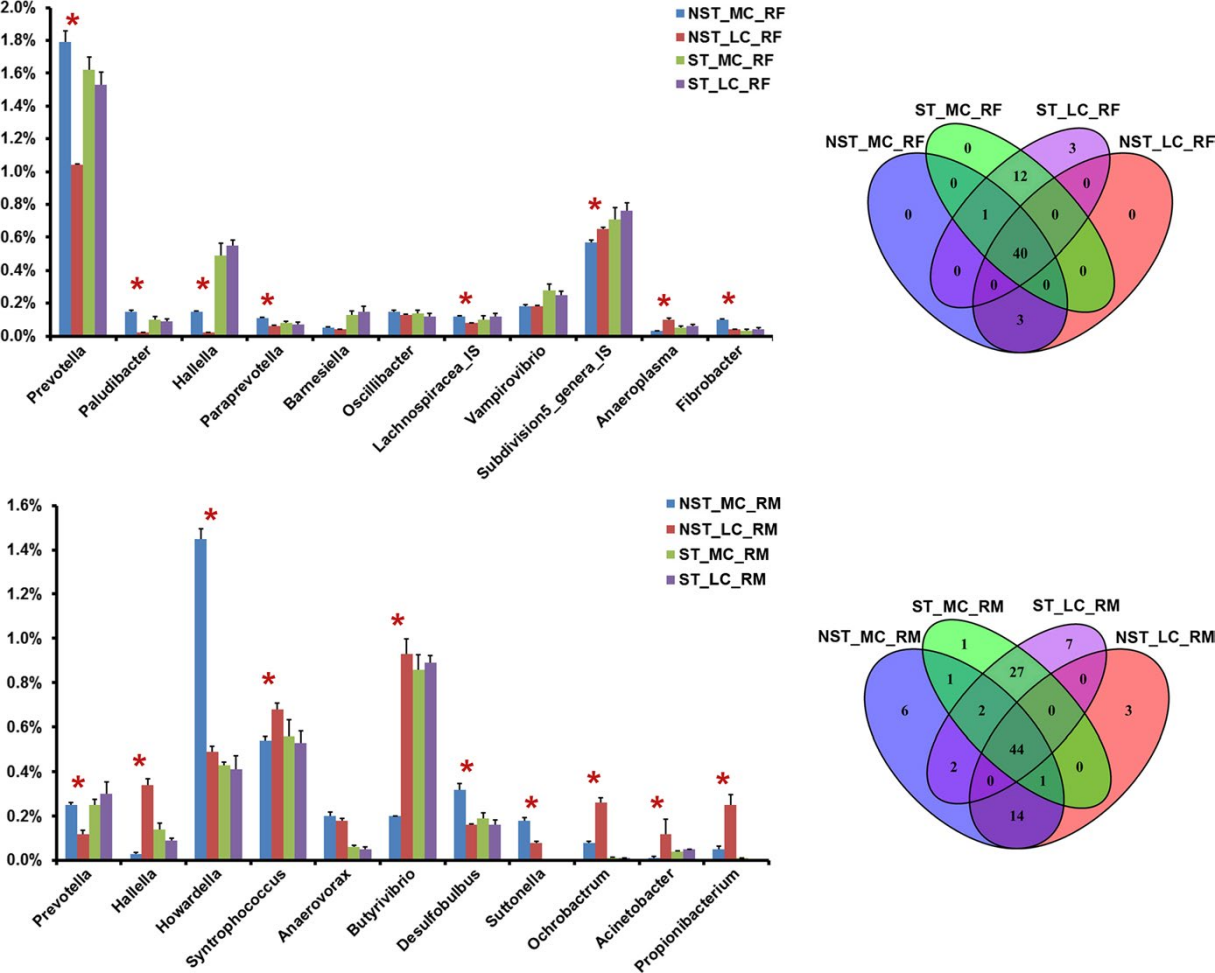
Genus‐level comparison of rumen fluid (RF) microbiota and rumen mucosal (RM) microbiota between samples from goats without exposure to the antibiotic and samples from goats with antibiotic treatment. Only the detectable genera (the relative abundance was more than 0.1% in at least one of the given samples) were present here. The genera were organized according to the phylum. Star indicates that significant differences are observed in the *t*‐test (*p *<* *.05). Lachnospiracea_IS indicates the candidate genus in family Lachnospiracea, and Subdivision_genera_IS indicates the candidate genus in phylum Verrucomicrobia

The long‐lasting (66 d) effect of antibiotic on rumen microbiota was revealed by a comparison of microbial structure between the NST_LC and ST_LC groups (Figures 2 and 3).

**Figure 4:**
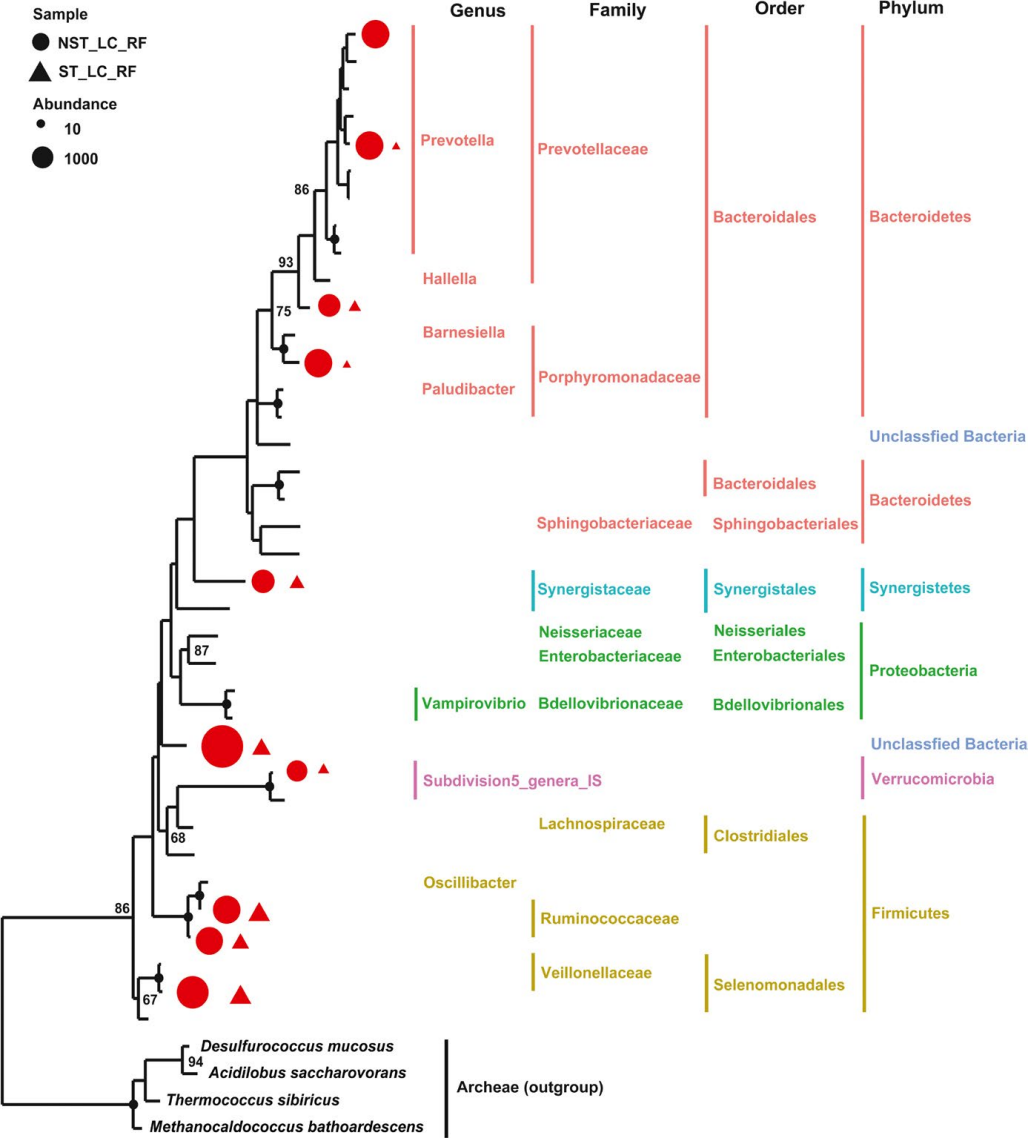
Maximum likelihood tree of 37 detectable OTUs (the relative abundance > 1% in at least one of the given samples) observed in rumen fluid (RF) microbiota. The received 16S *rRNA* gene sequences of the corresponding OTUs were used to construct the tree. Four archeaes were used as the outgroups. Triangle indicates the OTUs observed in goats with antibiotic treatment, and the circle indicates the OTUs observed in goats without exposure to the antibiotic. Only the OTUs with significant differences (*p *<* *.05) of relative abundance are shown behind the branches. The size of the symbol indicates the relative abundance of OTUs. Red indicates a significant decrease (*p *<* *.05) in the relative abundance of the OTU in goats with antibiotic treatment. Only those bootstrap values greater than 60 are shown on the tree. The solid black circles at the nodes stand for a bootstrap value of 100

For the microbial communities present in the rumen fluid (referred to as the NST_LC_RF and ST_LC_RF groups), a total of 14 phyla were found at the phylum level. Among them, after ST treatment, the relative abundances of four phyla (Bacteroidetes, Verrucomicrobia, Proteobacteria, and Armatimonadetes) were significantly higher, and the relative abundances of five phyla (Firmicutes, Tenericutes, Spirochaetes, Actinobacteria, and Chloroflexi) were significantly lower. Moreover, in total, 56 genera were found at the genus level, except for unclassified genera in the samples (Table S1). Among six detectable genera (detectable is defined as a relative abundance >1% in at least one sample in this study), Lachnospiracea_IS (candidate genus in family Lachnospiracea) was the most expanded genus, as its relative abundance increased by 100% after ST treatment. On the contrary, *Anaeroplasma* was the most reduced genus of all detectable genera, as its relative abundance decreased by 43% after ST treatment. Among 37 detectable operational taxonomic units (OTUs), the relative abundances of 4 OTUs belonging to Bacteroidetes, 3 OTUs belonging to Firmicutes, 1 OTU belonging to Verrucomicrobia, 1 OTU belonging to Synergistetes, and 1 unclassified OTU were significantly lower after ST treatment. However, none of OTUs was significantly higher after ST treatment (Figure 4; Figure S1).

**Figure 5:**
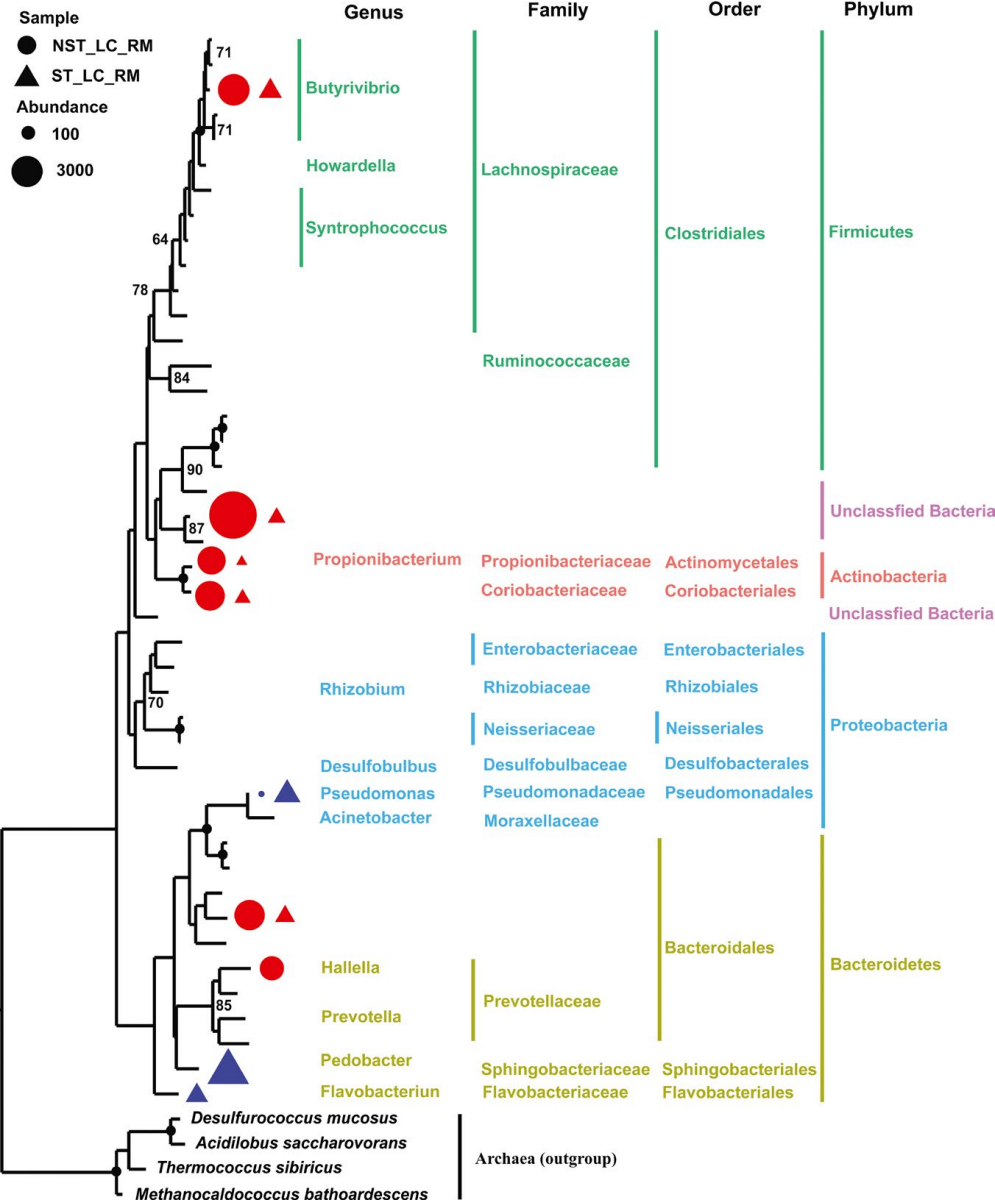
Maximum likelihood tree of 43 detectable OTUs (the relative abundance >1% in at least one of the given samples) observed in rumen mucosal (RM) microbiota. The received 16S rRNA gene sequences of the corresponding OTUs were used to construct the tree. Four archeaes were used as the outgroups. Triangle indicates the OTUs observed in goats with antibiotic treatment, and the circle indicates the OTUs observed in goats without exposure to the antibiotic. Only the OTUs with significant differences (*p *<* *.05) of relative abundance are shown behind the branches. The size of the symbol indicates the relative abundance of OTUs. Red indicates a significant decrease (*p *<* *.05) in the relative abundance of the OTU in goats with antibiotic treatment. Blue indicates a significant increase (*p *<* *.05) in the relative abundance of the OTU in goats with antibiotic treatment. Only those bootstrap values greater than 60 are shown on the tree. The solid black circles at the nodes stand for a bootstrap value of 100

For the microbial communities present in the rumen mucosal layer (referred to as the NST_LC_RM and ST_LC_RM groups), a total of 16 prokaryotic phyla were identified at the phylum level, and 12 were common to both groups. Firmicutes (48.2%–46.3%), Bacteroidetes (15.0%–21.7%), and Proteobacteria (14.6%–23.9%) were the most abundant phyla in these samples. Armatimonadetes was only observed in the NST_LC_RM group. Gemmatimonadetes, Elusimicrobia, and Planctomycetes were only observed in the ST_LC_RM group. Among 12 commonly observed phyla, the relative abundances of four phyla (Bacteroidetes, Proteobacteria, Saccharibacteria, and Tenericutes) were significantly higher, and the relative abundances of four phyla (Actinobacteria, Fibrobacteres, Chloroflexi, and Synergistetes) were significantly lower after ST treatment. Moreover, in total, 99 genera were found at the genus level, except for unclassified genera in the samples (Table S1). Among 10 detectable genera, *Pseudomonas* was the most expanded genus, as its relative abundance increased by 96 times after ST treatment. On the contrary, *Propionibacterium* and *Ochrobactrum* were the most reduced genera of all detectable genera, as their relative abundance decreased by 98% and 96% after ST treatment, respectively. *Pedobacter* and *Flavobacterium* were only detected in the ST_LC_RM group. Among 43 detectable OTUs, the relative abundances of 2 OTUs belonging to Bacteroidetes and 1 OTU belonging to Proteobacteria were significantly lower after ST treatment. Furthermore, the relative abundance of 1 OTU belonging to Firmicutes, 2 OTUs belonging to Actinobacteria, 2 OTUs belonging to Bacteroidetes, and 1 unclassified OTU were significantly higher after ST treatment (Figure 5; Figure S1).

### 3.2 Antibiotic pretreatment weakened effects of dietary concentrate on rumen microbiota reconstruction

The effect of dietary concentrate on the rumen microbiota of goats without ST treatment was revealed by a comparison of the microbial structure between the NST_LC and NST_MC groups. The effect of dietary concentrate on rumen microbiota of goats with ST treatment was revealed by a comparison of the microbial structure between the ST_LC and ST_MC groups. Figures 2 and 3 show the differences in the compositional alterations on the detectable phyla and genera.

For the RF microbiota in goats without ST treatment (NST_MC_RF and NST_LC_RF groups), 9 of 14 observed phyla showed significant changes after 4‐week MC feeding. Among them, Fibrobacteres increased by 185%, Lentisphaerae increased by 164%, and Proteobacteria increased by 46%, being the most expanded phyla after 4‐week MC feeding. Moreover, Tenericutes decreased by 77% and represented the most reduced phylum after 4‐week MC feeding. On the other hand, in the RF microbiota of ST‐treated goats (ST_MC_RF and ST_LC_RF groups), only the relative abundance of Proteobacteria was significantly lower after 4‐week MC feeding. On the genus level, after 4‐week MC feeding, 29 of 44 observed genera showed significant changes in goats without ST treatment. Among them, *Paludibacter* and *Hallella* increased by 738% and 508%, being the most expanded genera after 4‐week MC feeding. Furthermore, *Anaeroplasma* decreased by 73%, being most reduced genus after 4‐week MC feeding. On the other hand, in ST‐treated goats, no genus exhibited significant changes after 4‐week MC feeding. At the OTU level, 27 of 28 detectable OTUs showed significant changes in goats without ST treatment after 4‐week MC feeding (Figure S1). Among them, the relative abundances of 11 OTUs were significantly lower, and the relative abundances of 16 OTUs were significantly higher after 4‐week MC feeding. On the other hand, in ST‐treated goats, none of OTUs showed a significant change after 4‐week MC feeding.

For the RM microbiota in goats without ST treatment (NST_MC_RM and NST_LC_RM groups), 8 of 13 observed phyla showed significant changes after 4‐week MC feeding. Among them, Armatimonadetes increased by 224%, Spirochaetes increased by 202%, and Lentisphaerae increased by 123% after 4‐week MC feeding, being the most expanded phyla. Moreover, Actinobacteria decreased by 63% after 4‐week MC feeding, being the most shrunken phylum. On the other hand, in ST‐treated goats (ST_MC_RM and ST_LC_RM groups), Armatimonadetes was not observed; instead, Gemmatimonadetes, Acidobacteria, and Elusimicrobia occurred with low abundance. Furthermore, none of phyla showed a significant change after 4‐week MC feeding. On the genus level, 39 of 60 observed genera underwent significantly changes in goats without ST treatment after 4‐week MC feeding. Among them, *Howardella* increased by 196%, and *Prevotella* increased by 112%, being the most expanded genera. Moreover, both *Hallella* and *Acinetobacter* decreased by 92%, being the most shrunken genera after 4‐week MC feeding. On the other hand, in ST‐treated goats, no genus showed significant changes after 4‐week MC feeding. At the OTU level, 22 of 30 detectable OTUs exhibited significant changes in goats without ST treatment after 4‐week MC feeding (Figure S1). Among them, the relative abundances of 11 OTUs were significantly lower, and the relative abundances of another 11 OTUs were significantly higher, after ST treatment. On the other hand, in ST‐treated goats, none of OTUs revealed significant changes after 4‐week MC feeding.

### 3.3 Comparisons of diversity, richness, and structure of microbiota communities investigated in this study

Shannon and Simpson indices were calculated to evaluate the diversity of the investigated microbial communities (Figure S2). Accordingly, in goats without ST treatment, MC feeding led to the diversification of RF microbiota and simplification of RM microbiota. However, in goats underwent ST treatment, MC feeding did not induce significant changes in the diversity of either RF or RM microbiota.

Rarefaction curves were calculated to evaluate roughly the richness of the investigated samples. Accordingly, no matter whether goats underwent ST treatment or not, MC feeding led to the expansion of RF microbiota and the reduction in RM microbiota. However, ST pretreatment shortened the gap between the curves of MC and LC groups (Figure S3).

**Figure 6:**
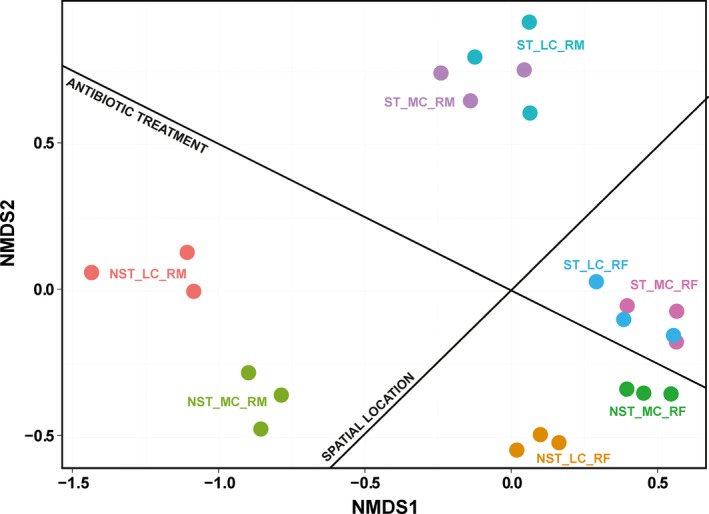
Nonmetric multidimensional scaling (NMDS) analysis of Bray‐Curtis similarity coefficients based on the relative abundance of OTUs of the corresponding samples

Nonmetric multidimensional scaling (NMDS) based on the Bray distance was calculated to evaluate the divergence of the community structure across the samples (Figure 6). Accordingly, spatial location and antibiotic treatment were two major factors affecting the structure of the microbial community. Diet affected the community structure in goats without ST treatment. However, it did not affect the community structure in goats underwent ST treatment.

### 3.4 Antibiotic pretreatment decreases the baselines of rumen fermentation products

No matter whether the goats underwent ST treatment or not, the concentrations of acetate, propinate, and total SCFA (TSCFA) were enhanced after 4–week MC feeding (*p *<* *.05). Moreover, the increased percentages of acetate and TSCFA concentrations were nearly the same between the NST and ST groups. However, a comparison of the concentrations of individual SCFAs and TSCFA before the dietary switch (baselines) revealed that all of them were lower in goats with ST treatment than those in goats without ST treatment (Table 1).

**Table 1:** Comparisons of short‐chain fatty acids (SCFAs) concentrations and gains between goats without exposure to the antibiotic and goats with antibiotic treatment

SCFAs	MC group (mM)			LC group (mM)			Mean gains (%)	
	NST_MC	ST_MC	*p* value^a^	NST_LC	ST_LC	*p* value^b^	NST [(MC‐LC)/LC]	ST [(MC‐LC)/LC]
Acetate	135.49 + 1.14	77.41 + 2.98	.01*	89.02 + 4.97	50.06 + 0.76	.03*	52.2%	54.6%
Propinate	22.15 + 0.51	17.31 + 1.38	.04*	17.60 + 1.14	12.77 + 0.69	.02*	25.9%	35.6%
Isobutyrate	1.03 + 0.06	0.91 + 0.04	.06	0.88 + 0.02	0.80 + 0.02	.13	17.0%	13.8%
Butyrate	8.65 + 0.48	7.24 + 0.53	.24	8.02 + 0.55	5.84 + 0.68	.19	7.9%	24.0%
Isovalerate	1.05 + 0.15	0.96 + 0.02	.54	0.84 + 0.06	0.83 + 0.02	.89	25.0%	15.7%
Valerate	0.82 + 0.10	0.80 + 0.06	.85	0.76 + 0.01	0.69 + 0.02	.12	7.9%	15.9%
Total SCAFA	166.29 + 0.55	101.96 + 3.72	.01*	114.65 + 6.09	68.67 + 0.61	.03*	45.0%	48.5%

a *p* value indicated the significance of paired *t*‐test between goats without exposure to the antibiotic receiving a MC diet during the dietary modulation experiment (NST_MC) group and goats with antibiotic treatment receiving a MC diet during the dietary modulation experiment (ST_MC) group.

b *p* value indicated the significance of paired *t*‐test between goats without exposure to the antibiotic receiving a LC diet during the dietary modulation experiment (NST_LC) group and goats with antibiotic treatment receiving a LC diet during the dietary modulation experiment (ST_LC) group.

*indicated the *p* value <.05

## 4 DISCUSSION

Recent studies have noted that heterogeneity exists between the fluid and mucosal microbiota of rumen. Our findings support the idea that a degree of segregation is present between the fluid and mucosal microbiota, and that such segregation is not affected by environmental stress, such as diet and antibiotics. Meantime, we have observed that intramuscular antibiotic injection induces alterations of both RF and RM microbiota. RM communities become diversified and expanded after ST treatment. Previous studies have indicated that environmental antibiotics, being an environmental pressure, promote bacteria to adhere to tissue surfaces by transforming their cells from free‐floating mode to the biofilm mode (Hoffman et al., 2005). In addition, previous investigations have revealed that the minimizing of the contact between the microbes and epithelial surface is the central strategy utilized by the host to maintain its homeostatic relationship with its microbiota (Belkaid & Hand, 2014). Therefore, we can reasonably infer that antibiotic treatment also disturbs the “healthy” interactions between the host and RM microbiota, leading to the gathering of microbes in the mucosal layer. On the contrary, RF communities are simplified and reduced after ST treatment. In this study, we thought that the alteration of RF microbiota might be induced by the injected antibiotic in an indirect way, because no glands have been observed in the rumen, and pharmacokinetic study show that only slight amounts of injected ST can be excreted into the saliva (Zhu et al., 2001). We therefore inferred that the alteration of RF microbiota was caused by the combined effects of the altered rumen environment, such as the increased secretion of antimicrobial components by the host and RM microbiota, and the migration of microbes to the mucosal surface. In addition, according to the ecological theory, if each species uses a slightly different nutrient resource and occupies a highly specific niche in the community, then the more diverse microbiota should be able to capture energy, and their resistance to invading pathogens is more efficient (Bell, Newman, Silverman, Turner, & Lilley, 2005). Since RF microbiota is the first line of defense against environmental pathogens, we inferred that the simplification and reduction in RF microbiota would enhance the pressure to the host immune system and thereafter aggravate the expansion and diversification of RM microbiota in the mucosal layer. However, further studies are required to clarify the interactions among the host and RF and RM microbiota.

Previous studies have revealed that patterns of microbiota alteration depend on the type of antibiotics used (Jernberg, Lofmark, Edlund, & Jansson, 2010). ST is an aminoglycosides drug. It inhibits the protein synthesis of both gram‐positive and gram‐negative bacteria. A recent study has shown that the presence of ST in the environment promotes the biofilm formation of specific bacteria (Kumar & Ting, 2016). Once the biofilm starts to form, its polysaccharide matrices shield the internal cells from environmental elements and, therefore, amplify bacterial resistance to antibiotics and the host's attack (Nadell, Xavier, & Foster, 2009). Previous studies have demonstrated that the development of a biofilm aggregates the different types of bacteria together to form biofilm communities. Community members can share genetic features using quorum‐sensing and internal horizontal gene transfer (Belkaid & Hand, 2014; Nadell et al., 2009). In the present study, *Prevotella*,* Pseudomonas, Pedobacter*, and *Flavobacterium* are significantly expanded in the RM community after ST treatment. Members of *Pseudomonas* have been shown to be the dominant members in many types of biofilm communities and have resistance to multidrugs (Heintz & Halilovic, 2010). Members of *Pedobacter* are able to produce antimicrobial compounds that inhibit the growth of food pathogens (Wong et al., 2011) and additionally possess the CRISPR‐Cas system, an adaptive immune system that defends against foreign genetic elements derived from bacteriophages (Poehlein, Daniel, & Simeonova, 2015). Accordingly, the colonization of these species in the mucosal layer benefits the formation and development of polymicrobial biofilms. Although the functions of most of the species in RM microbiota that had expanded after ST treatment were unclear, previous investigations have shown that the abundance of *Prevotella, Pseudomonas, Pedobacter*, and *Flavobacterium* are regularly high in biofilm communities associated with injured tissue. Accordingly, we have inferred that antibiotic treatment promotes the formation and development of polymicrobial biofilms in the mucosal layer, and that the biofilm gives competitive advantages to the members within the communities. In the dietary modulation experiment, the structure of recovered RM microbiota was not affected by the dietary shift, indicating that the community shaped by the antibiotic is highly stable.

In this study, the relative abundances of 10 OTUs in RF microbiota were significantly lower after ST treatment. Among them, members of the Ruminococcaceae are important contributors to cellulose degradation (Weimer, 1996). Members of the Veillonellaceae and Prophyromonadacea are capable of utilizing lactate and converting it largely to acetate and propionate (Duncan, Barcenilla, Stewart, Pryde, & Flint, 2002). Members of the Synergistaceae are able to ferment amino acids and peptides to produce acetate in animal gut (Leong, Denman, Hugenholtz, & McSweeney, 2016). Members of the *Prevotella* are able to ferment simple carbohydrates to produce propionate in the GI tract (Matsui et al., 2000). A recent study of the first cultured representative of Verrucomicrobia subdivision 5 indicates that this strain of the taxon ferments monosaccharide to produce acetate and lactate (Spring et al., 2016). Therefore, the reduction in these bacteria might explain the decreased baselines of SCFA products after ST treatment. Second, in the following experiment, the recovered RF microbiota was little affected by the dietary shift. However, in goats without ST treatment, the structure of RF microbiota was significantly affected by the dietary shift. These findings supported our previous hypothesis that the rumen environment most probably reshaped the RF microbiota. Next, the results also showed that the environmental factor that reshaped the RF microbiota during the antibiotic period did not disappear in the dietary modulation period, and moreover, that it was still the dominant factor in the shaping of RF microbiota in the dietary modulation period.

Previous studies have indicated that functional redundancy is an important feature for the rumen microbiota, and that it allows an altered community to perform ecosystem functions equivalent to those of the original community (Weimer, 2015). Rumen fermentation patterns, including acetate‐type fermentation (the ratio of acetate to propionate is higher than 3), and propionate‐type fermentation (the ratio of acetate to propionate is lower than 3), reflect the ratio of two major microbial populations, i.e., acetate‐producing bacteria and propionate‐producing bacteria, in the rumen microbiota, having the practical significance in livestock production. In this study, the molar percentages of individual SCFAs and the increased percentage of TSCFA upon the shift of diet exhibited no significant changes after ST treatment, indicating that the composition of the core members/functional genes of the rumen microbiota concerning the rumen fermentation are not affected by the antibiotic. However, the baselines of SCFAs are reduced after ST treatment, indicating that the redundancy of functional gene pool is reduced. Therefore, we inferred that, despite the differences in community membership, the basic gene content and core members of RF microbiota at the functional level are not affected or can recover from the antibiotic administration.

## 5 CONCLUSION

In this study, we used 16s *rRNA* gene sequencing to investigate the posteffect of ST administration on RF and RM microbiota in an *in vivo* condition. Our findings are summarized as follows. First, the simultaneous alteration of RM and RF microbiota was observed when the microenvironment of one community (intramuscular ST injection and dietary shift) was disturbed, suggesting that these communities are mutually interrelated. Second, antibiotic injection induced the increases in the diversity and richness of RM microbiota, coupled with the significant expansion of *Prevotella, Pseudomonas, Pedobacter*, and *Flavobacterium*, which are regular members of biofilm communities attached to injured tissue. This indicates that antibiotic injection disturbs the interaction of the host and commensals, leading to a gathering of biofilm communities in the mucosal layer. Third, the diversity and richness of RF microbiota are reduced in the ST‐pretreated goats, coupled with reduced baselines of major fermentation products in the rumen. These results suggest that the rumen environment recovers from ST administration but that this is not good for the growth of SCFA‐producing bacteria to a certain extent. Finally, dietary modulation in the subsequent experiment could not affect the structure of the recovered RF and RM microbiota or the rumen fermentation pattern and gained SCFAs under dietary modulation. This indicates that the redundant genes and members are reduced after antibiotic administration. However, the core members involved in rumen fermentation are not affected and are stable in the rumen. Altogether, our findings indicate that antibiotic administration reduces the genetic diversity of rumen microbiota, and that such a reduction is long‐lasting and cannot be easily compensated by the diet. Additionally, the alteration of the spatial organization observed in our study suggests that the modulation of rumen fermentation should aim to improve the rumen environment rather than the exclusive supplements of single prebiotics.
