# Supplementary material for: Antibiotic pretreatment minimizes dietary effects on reconstructure of rumen fluid and mucosal microbiota in goats
Source: Microbiologyopen. 2017 Oct 5;7(1):e00537. doi: 10.1002/mbo3.537 (PMC5822325; doi:10.1002/mbo3.537)
Supplement: Supplementary file 1 [file MBO3-7-na-s001.pdf]

Fig. S1. (A) OTU-level comparison of diet-induced alterations between antibiotic-pretreated rumen fluid (RF) microbiota and indigenous RF microbiota. (B) OTU-level comparison of diet-induced alterations between antibiotic-pretreated rumen mucosal (RM) microbiota and indigenous RM microbiota.

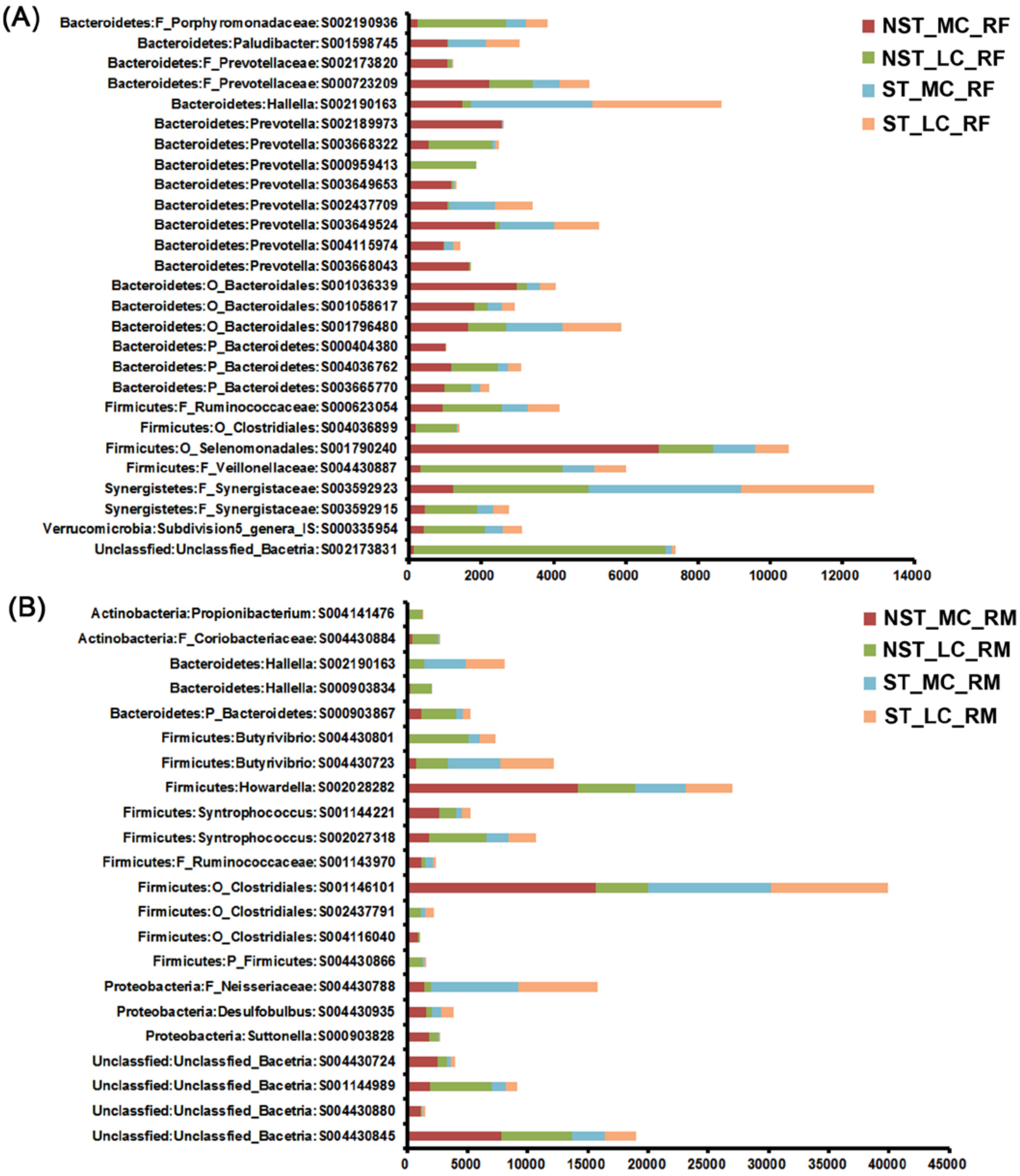

Fig. S2. Community diversity estimated by using Shannon and Simpson indices

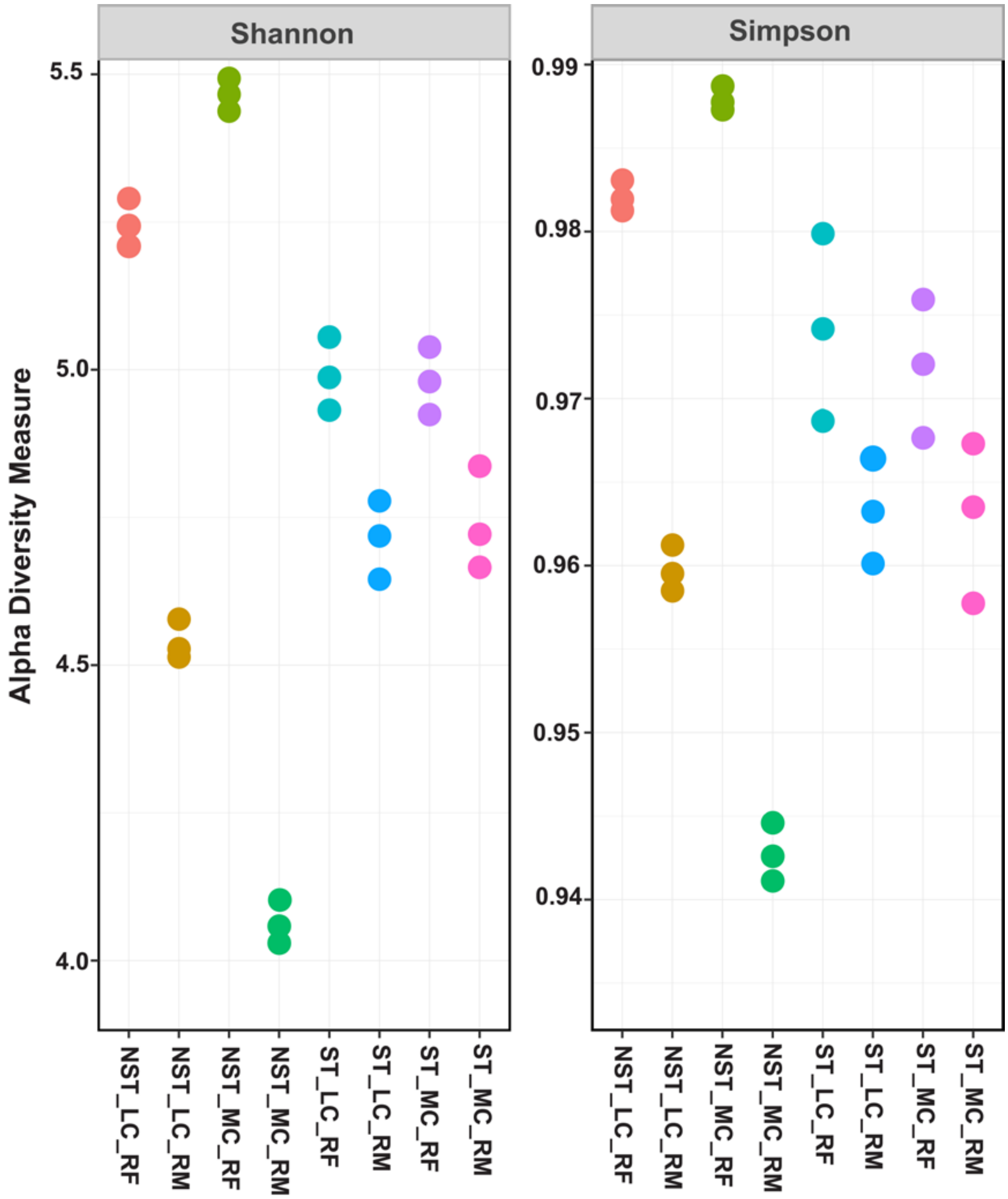

Fig. S3. Richness of rumen microbiota estimated by using rarefaction curves.

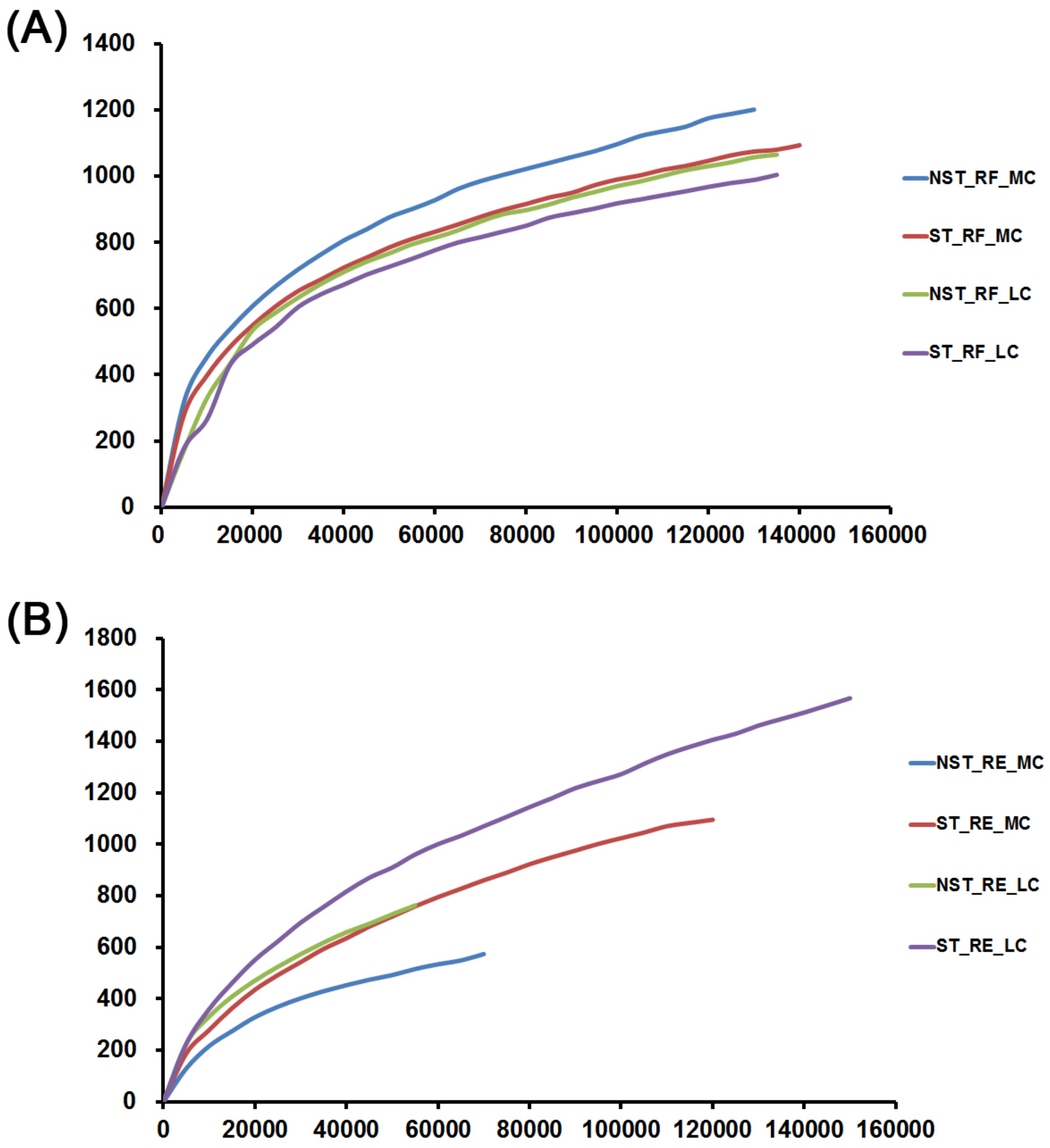

Table S1. Compositions of rumen fluid and mucosal microbiota (mean percentage ± SE; N = 3).

Item	Genus	NST_MC_RM	NST_MC_RF	NST_LC_RM	NST_LC_RF	ST_MC_RM	ST_MC_RF	ST_LC_RM	ST_LC_RF
1	Acetivibrio	98±41	304±18	10±10	254±16	104±20	251±56	148±44	267±60
2	Acidiferrobacter	0±0	0±0	0±0	0±0	18±12	0±0	13±7	0±0
3	Acidovorax	0±0	0±0	0±0	0±0	8±5	0±0	37±26	1±1
4	Acinetobacter	115±83	0±0	1316±675	0±0	424±53	30±7	1497±1114	43±38
5	Actinomycetospora	14±14	0±0	0±0	0±0	0±0	0±0	0±0	0±0
6	Akkermansia	0±0	17±4	0±0	0±0	1±1	0±0	0±0	0±0
7	Allofustis	0±0	0±0	0±0	0±0	1±1	1±1	6±5	4±0
8	Alloprevotella	14±14	64±3	175±104	0±0	312±152	11±8	194±187	58±46
9	Anaerofustis	43±43	2±2	0±0	18±3	19±5	12±5	14±7	6±3
10	Anaeroplasma	45±35	292±19	105±62	1037±77	401±132	541±319	239±127	592±208
11	Anaerorhabdus	11±11	3±2	55±55	8±1	4±4	1±1	0±0	4±2
12	Anaerovibrio	0±0	31±8	0±0	49±4	0±0	24±10	0±0	21±2
13	Anaerovorax	2031±204	887±47	1791±106	708±45	578±90	512±21	488±115	488±39
14	Armatimonadetes_gp2	7±7	94±11	10±10	12±9	6±3	58±23	1±1	37±27
15	Armatimonadetes_gp5	54±54	0±0	17±17	0±0	6±6	0±0	0±0	0±0
16	Atopobium	699±61	79±1	747±111	48±13	375±49	40±13	419±103	43±18
17	Azomonas	0±0	0±0	0±0	0±0	13±5	0±0	25±12	0±0
18	Bacillus	0±0	0±0	0±0	0±0	53±12	0±0	104±67	5±5
19	Barnesiella	172±25	454±39	155±82	373±25	447±153	734±627	260±184	1539±703
20	Bdellovibrio	11±11	0±0	0±0	0±0	0±0	0±0	7±7	0±0

Item	Genus	NST_MC_RM	NST_MC_RF	NST_LC_RM	NST_LC_RF	ST_MC_RM	ST_MC_RF	ST_LC_RM	ST_LC_RF
21	Bilophila	0±0	23±5	0±0	0±0	0±0	0±0	0±0	0±0
22	Blastobacter	0±0	0±0	0±0	0±0	0±0	0±0	6±4	0±0
23	Blautia	49±12	6±6	9±9	19±3	48±16	31±5	9±4	52±10
24	Buttiauxella	0±0	0±0	22±22	0±0	2±2	0±0	0±0	0±0
25	Butyricicoccus	0±0	0±0	0±0	0±0	16±5	0±0	35±15	3±3
26	Butyrivibrio	1992±19	697±33	9202±659	786±30	5542±4637	303±53	8844±4291	295±52
27	Candidatus_Endomicrobium	5±5	26±7	0±0	36±3	8±8	51±9	9±8	22±11
28	Chelatococcus	82±49	2±2	170±78	0±0	0±0	0±0	0±0	0±0
29	Clostridium_IV	108±56	214±13	138±77	838±57	218±22	580±56	242±43	780±46
30	Clostridium_XlVa	64±40	69±6	61±35	103±23	103±19	92±34	174±96	142±36
31	Clostridium_XlVb	369±52	4±2	188±66	0±0	267±215	2±1	497±250	2±2
32	Clostridium_XVIII	0±0	0±0	0±0	0±0	11±7	0±0	16±11	0±0
33	Coprococcus	90±30	44±8	0±0	39±5	48±16	21±14	18±15	31±17
34	Desulfobulbus	3138±257	19±10	1635±48	16±5	1911±725	54±19	1932±669	53±17
35	Desulfovibrio	0±0	93±16	0±0	79±4	11±6	64±25	11±6	108±34
36	Diplorickettsia	30±30	0±0	49±49	0±0	0±0	0±0	0±0	0±0
37	Ensifer	101±12	0±0	316±148	2±2	48±11	1±1	50±7	8±8
38	Enterobacter	0±0	0±0	0±0	0±0	0±0	0±0	9±9	0±0
39	Enterorhabdus	102±17	13±4	31±31	17±6	15±5	14±8	10±5	8±2
40	Escherichia	2±2	0±0	0±0	0±0	213±27	0±0	548±346	16±11
41	Eubacterium	430±14	6±1	249±125	0±0	121±115	4±2	221±111	4±2
42	Fibrobacter	647±69	1041±62	935±147	381±40	275±20	268±131	236±79	378±137

Item	Genus	NST_MC_RM	NST_MC_RF	NST_LC_RM	NST_LC_RF	ST_MC_RM	ST_MC_RF	ST_LC_RM	ST_LC_RF
43	Filifactor	0±0	0±0	0±0	0±0	43±18	22±10	44±14	26±9
44	Flavobacterium	0±0	0±0	0±0	0±0	423±125	13±1	1416±887	34±29
45	Flavonifractor	5±5	0±0	0±0	2±2	15±5	0±0	24±20	6±4
46	Gemmatimonas	0±0	0±0	0±0	0±0	24±12	0±0	14±8	0±0
47	Gp6	0±0	0±0	0±0	0±0	13±13	0±0	0±0	0±0
48	Hallella	280±64	1495±16	3378±280	244±17	4415±1987	2920±1700	2239±1886	5484±2329
49	Helcococcus	0±0	0±0	0±0	0±0	7±4	0±0	7±4	1±1
50	Howardella	14371±466	30±2	4854±263	36±6	4321±129	34±12	3522±559	294±280
51	Hymenobacter	0±0	0±0	0±0	0±0	0±0	0±0	5±5	0±0
52	Lachnoanaerobaculum	5±5	3±3	0±0	9±6	4±4	13±4	11±8	18±5
53	Lachnospiracea_IS	139±16	1184±47	83±44	822±18	124±21	1012±556	124±32	1647±581
54	Leucothrix	0±0	0±0	0±0	0±0	12±6	0±0	5±3	0±0
55	Microbacterium	49±1	1±1	82±47	0±0	4±2	0±0	3±2	1±1
56	Mogibacterium	46±21	19±7	14±8	12±9	8±5	7±5	14±12	5±3
57	Moryella	31±31	28±2	10±10	63±10	31±15	14±5	38±23	21±9
58	Murdochiella	0±0	0±0	0±0	0±0	16±11	1±1	4±4	0±0
59	Nocardioides	6±3	0±0	118±61	0±0	0±0	0±0	0±0	0±0
60	Novosphingobium	11±11	0±0	0±0	0±0	4±4	0±0	1±1	0±0
61	Ochrobactrum	805±70	2±2	2633±929	3±3	176±49	0±0	116±9	22±20
62	Odoribacter	0±0	0±0	0±0	0±0	11±6	0±0	14±12	5±3
63	Ohtaekwangia	0±0	0±0	0±0	0±0	10±5	0±0	6±6	0±0
64	Oligosphaera	34±29	321±22	38±38	167±22	27±16	416±153	31±15	292±154

Item	Genus	NST_MC_RM	NST_MC_RF	NST_LC_RM	NST_LC_RF	ST_MC_RM	ST_MC_RF	ST_LC_RM	ST_LC_RF
65	Olsenella	138±52	17±7	3±3	50±3	35±16	23±9	33±13	11±3
66	Opitutus	19±19	0±0	0±0	0±0	0±0	0±0	0±0	0±0
67	Oscillibacter	70±35	1490±51	102±62	1292±10	72±7	1373±176	112±32	1218±159
68	Paludibacter	18±9	1520±57	31±31	181±45	80±42	1016±163	156±72	741±157
69	Paraprevotella	163±76	1108±22	33±21	569±40	107±58	748±75	125±36	711±141
70	Patulibacter	16±16	0±0	0±0	0±0	0±0	0±0	0±0	0±0
71	Pedobacter	0±0	2±2	0±0	0±0	2093±410	43±12	4899±3486	122±111
72	Pelomonas	0±0	0±0	0±0	0±0	21±9	0±0	13±10	0±0
73	Peptococcus	0±0	0±0	0±0	0±0	6±6	0±0	6±6	0±0
74	Phaselicystis	0±0	0±0	0±0	0±0	0±0	0±0	4±4	0±0
75	Phycisphaera	0±0	0±0	0±0	0±0	0±0	0±0	8±6	0±0
76	Phyllobacterium	0±0	0±0	36±31	0±0	0±0	0±0	0±0	0±0
77	Prevotella	2610±137	17808±679	1236±154	10391±31	2536±1262	22127±8260	3385±1207	12328±7632
78	Propionibacterium	476±159	9±5	2494±465	28±11	78±28	0±0	50±9	3±1
79	Pseudobutyrivibrio	0±0	51±9	0±0	19±7	0±0	0±0	0±0	0±0
80	Pseudochrobactrum	32±13	0±0	108±49	0±0	4±4	0±0	3±0	0±0
81	Pseudoflavonifractor	0±0	0±0	0±0	0±0	0±0	0±0	33±31	0±0
82	Pseudomonas	20±12	0±0	79±7	0±0	4006±657	85±19	8353±5585	344±298
83	Pseudonocardia	24±12	0±0	27±27	0±0	0±0	0±0	0±0	0±0
84	Pseudoramibacter	0±0	4±4	0±0	0±0	0±0	2±2	13±2	0±0
85	Rhizobium	143±27	1±1	480±109	8±4	270±79	14±3	687±488	24±20
86	Rhodoplanes	0±0	0±0	0±0	0±0	8±8	0±0	0±0	0±0

Item	Genus	NST_MC_RM	NST_MC_RF	NST_LC_RM	NST_LC_RF	ST_MC_RM	ST_MC_RF	ST_LC_RM	ST_LC_RF
87	Rhodovulum	14±14	0±0	0±0	0±0	0±0	0±0	0±0	0±0
88	Roseburia	0±0	104±13	27±27	69±8	2±2	5±1	0±0	4±2
89	Roseomonas	0±0	0±0	0±0	0±0	5±4	0±0	5±5	0±0
90	Ruminobacter	0±0	2±2	0±0	0±0	0±0	22±11	8±6	10±10
91	Ruminococcus	279±63	486±41	229±38	522±49	154±8	534±79	145±40	667±59
92	Ruminococcus2	0±0	0±0	0±0	0±0	0±0	0±0	5±5	0±0
93	Saccharibacteria_genera_IS	63±11	828±34	219±46	775±51	422±352	471±23	580±280	416±33
94	Saccharofermentans	63±9	250±32	17±17	121±21	38±12	44±20	44±14	52±15
95	Schwartzia	19±10	319±15	27±27	300±35	18±18	331±117	42±15	265±155
96	Selenomonas	5±5	5±3	0±0	6±3	0±0	11±8	2±2	12±5
97	Serratia	0±0	0±0	0±0	0±0	14±5	0±0	48±26	9±9
98	Slackia	6±3	376±16	77±43	205±21	4±4	83±37	12±5	58±19
99	Solirubrobacter	33±19	0±0	132±132	0±0	0±0	0±0	0±0	0±0
100	Sphaerobacter	64±47	0±0	571±217	0±0	45±22	0±0	17±13	0±0
101	Sphaerochaeta	22±12	38±2	11±11	298±14	6±6	285±146	28±21	148±142
102	Sphingomonas	85±44	0±0	208±51	0±0	0±0	0±0	0±0	0±0
103	Spirosoma	14±14	0±0	16±16	0±0	0±0	0±0	3±3	0±0
104	Sporobacter	52±12	154±2	20±20	56±4	96±34	128±32	79±41	74±37
105	Staphylococcus	103±54	0±0	631±207	0±0	6±6	0±0	4±3	0±0
106	Streptococcus	13±13	6±3	253±182	12±12	0±0	0±0	0±0	1±1
107	Subdivision3_genera_IS	0±0	0±0	0±0	0±0	2±2	0±0	8±4	0±0
108	Subdivision5_genera_IS	276±17	5682±136	245±168	6470±95	256±20	7078±715	218±60	7571±490

Item	Genus	NST_MC_RM	NST_MC_RF	NST_LC_RM	NST_LC_RF	ST_MC_RM	ST_MC_RF	ST_LC_RM	ST_LC_RF
109	Succiniclasticum	17±12	40±15	0±0	51±8	8±2	46±7	2±2	30±6
110	Succinivibrio	6±3	3±2	0±0	8±1	1±1	12±10	0±0	11±6
111	Suttonella	1829±124	21±11	762±50	7±4	44±22	1±1	35±34	22±22
112	Syntrophococcus	5391±192	231±15	6782±273	141±16	5544±3884	174±55	8057±3644	236±94
113	Thermus	27±27	0±0	20±10	0±0	0±0	0±0	0±0	0±0
114	Treponema	451±80	864±36	243±86	575±17	298±9	428±150	208±59	492±143
115	Vampirovibrio	146±25	1825±92	332±68	1830±72	154±38	2762±357	117±8	2497±234
116	Variovorax	11±11	0±0	49±49	0±0	0±0	0±0	0±0	0±0
117	Victivallis	56±33	953±21	0±0	316±11	2±2	595±218	17±15	462±270
118	C_Acidobacteria_Gp4	0±0	0±0	0±0	0±0	8±8	0±0	0±0	0±0
119	C_Actinobacteria	11±11	0±0	0±0	0±0	0±0	0±0	0±0	0±0
120	C_Alphaproteobacteria	275±76	1544±78	442±148	363±18	132±80	1512±143	195±74	1091±115
121	C_Betaproteobacteria	0±0	8±3	24±24	9±5	80±24	16±11	35±28	15±6
122	C_Clostridia	15±12	57±8	43±35	136±13	20±4	71±5	12±5	75±13
123	C_Deltaproteobacteria	48±30	452±22	217±57	126±19	111±25	225±65	129±16	306±84
124	C_Gammaproteobacteria	0±0	0±0	0±0	0±0	8±8	0±0	0±0	0±0
125	F_Acetobacteraceae	0±0	0±0	0±0	0±0	171±83	0±0	47±25	0±0
126	F_Anaerolineaceae	266±44	48±15	113±66	209±24	24±24	152±59	40±4	85±33
127	F_Aurantimonadaceae	22±20	0±0	29±11	0±0	0±0	0±0	1±1	0±0
128	F_Bifidobacteriaceae	0±0	0±0	0±0	0±0	4±4	2±2	2±2	0±0
129	F_Bradyrhizobiaceae	0±0	0±0	0±0	0±0	18±12	0±0	3±2	0±0
130	F_Burkholderiales_IS	0±0	0±0	0±0	0±0	12±9	0±0	0±0	0±0

Item	Genus	NST_MC_RM	NST_MC_RF	NST_LC_RM	NST_LC_RF	ST_MC_RM	ST_MC_RF	ST_LC_RM	ST_LC_RF
131	F_Chloroflexaceae	0±0	0±0	0±0	0±0	33±17	0±0	18±9	0±0
132	F_Clostridiales_IS_XIII	431±70	0±0	0±0	2±2	68±35	4±2	31±31	10±10
133	F_Comamonadaceae	10±3	0±0	22±6	0±0	5±4	0±0	11±4	0±0
134	F_Coriobacteriaceae	580±44	486±13	2074±283	546±29	239±10	269±113	161±30	179±70
135	F_Desulfovibrionaceae	186±25	174±29	99±80	432±12	89±13	219±122	69±10	293±104
136	F_Enterobacteriaceae	3538±317	307±26	4616±494	155±28	5780±1898	232±89	3792±2335	651±374
137	F_Erysipelotrichaceae	230±23	155±14	67±43	1039±45	55±12	156±21	47±18	120±18
138	F_Lachnospiraceae	7261±338	3487±49	8362±285	5901±54	4787±1863	3424±486	6246±2081	3700±403
139	F_Microbacteriaceae	0±0	0±0	0±0	0±0	10±10	0±0	0±0	0±0
140	F_Mycoplasmataceae	0±0	0±0	0±0	180±21	4±4	23±8	31±25	13±5
141	F_Neisseriaceae	1705±94	8±1	505±86	15±4	9571±3811	77±34	5204±3680	1415±1349
142	F_Peptostreptococcaceae	0±0	15±5	0±0	0±0	0±0	48±25	9±5	17±13
143	F_Phyllobacteriaceae	33±6	0±0	89±54	0±0	2±2	0±0	6±2	0±0
144	F_Porphyromonadaceae	774±115	1420±26	451±47	4812±164	734±47	2429±548	685±146	2957±590
145	F_Prevotellaceae	815±120	4125±77	904±108	2777±86	5326±2388	1578±508	2696±2327	2171±248
146	F_Pseudomonadaceae	0±0	0±0	5±5	0±0	299±62	7±4	565±375	26±21
147	F_Puniceicoccaceae	0±0	15±7	0±0	4±4	0±0	6±4	2±1	6±4
148	F_Rhodocyclaceae	0±0	0±0	0±0	0±0	8±8	0±0	2±2	0±0
149	F_Rhodospirillaceae	16±16	0±0	0±0	0±0	20±10	0±0	4±4	0±0
150	F_Ruminococcaceae	3492±191	5405±116	3240±269	6342±170	4219±581	5657±129	3597±875	5320±237
151	F_Sphingobacteriaceae	0±0	249±14	0±0	2±2	131±60	3318±3265	82±71	5029±2486
152	F_Spirochaetaceae	542±33	192±30	99±75	254±18	99±56	143±15	175±76	134±28

Item	Genus	NST_MC_RM	NST_MC_RF	NST_LC_RM	NST_LC_RF	ST_MC_RM	ST_MC_RF	ST_LC_RM	ST_LC_RF
153	F_Sutterellaceae	0±0	41±6	0±0	0±0	2±2	17±6	0±0	7±4
154	F_Synergistaceae	315±143	6914±41	511±49	10884±121	243±114	11135±3164	331±108	9811±3316
155	F_Veillonellaceae	354±49	2956±93	134±42	6542±184	105±20	2344±283	142±34	2501±387
156	O_Actinomycetales	0±0	0±0	0±0	0±0	18±18	0±0	15±11	0±0
157	O_Bacillales	0±0	2±2	0±0	0±0	126±42	2±2	230±133	13±9
158	O_Bacteroidales	2181±206	10318±164	4213±255	4161±68	2970±969	5546±609	3354±928	5021±341
159	O_Burkholderiales	0±0	0±0	0±0	10±3	17±6	7±5	19±5	18±13
160	O_Campylobacterales	119±30	98±12	105±15	116±10	121±31	195±23	110±15	199±20
161	O_Chromatiales	14±14	2±2	53±48	0±0	0±0	0±0	0±0	0±0
162	O_Clostridiales	19324±185	2602±46	8897±610	4770±79	16806±4252	2936±484	11461±4780	3239±466
163	O_Desulfuromonadales	51±51	33±11	4±4	38±7	63±43	37±3	89±53	35±4
164	O_Gammaproteobacteria_IS	0±0	0±0	0±0	0±0	22±19	0±0	3±2	0±0
165	O_Myxococcales	0±0	0±0	0±0	0±0	60±26	0±0	16±8	0±0
166	O_Rhizobiales	7±7	0±0	27±27	0±0	17±6	0±0	9±6	0±0
167	O_Rhodospirillales	0±0	0±0	214±214	0±0	40±23	0±0	11±6	0±0
168	O_Selenomonadales	64±30	6853±59	223±37	1475±38	24±21	1173±462	43±24	724±410
169	P_Bacteroidetes	2820±41	4677±108	4330±192	3010±39	2704±503	4171±2068	2121±554	5249±1871
170	P_Chloroflexi	119±3	0±0	58±39	2±2	0±0	0±0	0±0	0±0
171	P_Firmicutes	1023±148	2223±126	2497±342	3494±43	1459±58	2399±907	1342±169	2402±589
172	P_Proteobacteria	7±5	32±9	0±0	14±7	16±7	60±29	5±1	77±19
173	Unclassfied_Bacetria	14250±536	3343±100	13217±1074	11668±133	5413±231	3822±1600	5259±681	5713±1891

Counts were normalized to the relative abundance of each sample and showed here in a scale of 1/100,000. F = family; O = order; C = class; P = phylum. IS: incertae

sedis

Table S2. Dietary compositions used in this study

Ingredients	MC (%)	LC (%)
Guinea grass	65	90
Corn	25	0
Soya bean meal	8	8
Additive	2	2

The additive was composed of calcium phosphate, limestone, trace mineral salt, and vitamin premix (vitamins A, D, and E).
